# Response to: Lagunes-Cordoba et al ‘International medical graduates: how can UK psychiatry do better?’

**DOI:** 10.1192/bjb.2022.12

**Published:** 2022-04

**Authors:** Paul Tiffin, Lewis Paton

**Affiliations:** Professor of Health Services and Workforce Research, University of York, UK. Email: paul.tiffin@york.ac.uk; Lecturer in Data Science, University of York, UK

The paper by Lagunes-Cordoba et al^1^ makes important points in relation to differential attainment in psychiatry. However, we note the statement: ‘we note that technically the term “IMG” applies to a White British citizen who studies abroad and returns to work in the UK, yet such an individual is less likely to face attainment gaps’. This may not be entirely true, depending on what one means by ‘attainment’ in this context. We previously published a study using data drawn from the UK Medical Education Database (UKMED), which investigated educational performance and success at recruitment into specialty training for UK International Medical Graduates (IMGs). These are doctors who are UK citizens but have obtained their primary medical qualification outside the UK. We showed that, on average, ratings at the Annual Review of Competence Progression were poorer for UK IMGs than non-UK IMGs. Nevertheless, UK IMGs were more likely to be successful, compared with IMGs, when applying for a specialty training post.^2^ This finding obviously raises issues of fairness, and effectiveness, in postgraduate medical selection. We would also wish to draw attention to our own recently published study of differential attainment in the MRCPsych examination, which was not cited in the paper, though highly relevant.^3^ This demonstrated that differential pass rates at the Clinical Assessment of Skills and Competencies existed for candidates (both UK graduates and IMGs) who identified as being from minority ethnic groups, even after controlling for the influence of performance on knowledge-based components of the examination. Similar findings were previously reported by Esmail, for the Clinical Skills Assessment component of the MRCGP.^4^ At the time we suggested that these differential pass rates were likely to have complex underlying causes but urgently required investigating and addressing. Understanding and addressing differential attainment is clearly a matter of social justice but is also essential to the well-being of the National Health Service, its patients and the overseas-qualified staff it has traditionally relied on. Therefore, we felt it was important to draw attention to our own findings, which we believe have contributed to understanding this important but sensitive area of workforce research.
